# An Extremely Rare Anomaly: Unveiling Renal Vein-Originated Leiomyosarcoma

**DOI:** 10.1155/cris/1335881

**Published:** 2025-06-02

**Authors:** Jihane El Hamzaoui, Ali Kada, Imane El Messaoudi, Fouad Zouaidia, Hamza Sekkat, Youness Bakali, Mouna Mhamdi Alaoui, Farid Sabbah, Abdelmalek Hrora, Mohammed Raiss

**Affiliations:** ^1^Department of General Surgery C, Centre Hospitalier Ibn Sina, Rabat, Morocco; ^2^Faculty of Medicine and Pharmacy, Mohammed V University in Rabat, Rabat, Morocco; ^3^Anatomopathological Department, Ibn Sina University Hospital, Rabat, Morocco

**Keywords:** abdominal mass, leiomyosarcoma, renal vein

## Abstract

**Introduction:** Angiogenic leiomyosarcoma (LMS), a soft tissue sarcoma, primarily occurs in the inferior vena cava (IVC) in over 50% of cases, with renal vein LMSs being exceedingly rare. We present a case of primary LMS of the left renal vein.

**Case Report:** A 73-year-old woman with a history of hypertension and prior left colon adenocarcinoma presented with intermittent left flank pain. Imaging revealed a large left latero–aortic mass. Exploratory laparotomy confirmed a multinodular tumor around the left renal hilum, necessitating en bloc resection with left nephrectomy. Pathological examination identified it as a grade 2 LMS. The patient recovered well postoperatively with no complications.

**Discussion:** LMSs, especially of vascular origin, are rare and aggressive malignancies. Despite their insidious presentation, they predominantly manifest in women, typically adults, and often on the left side. Diagnosis is challenging due to nonspecific symptoms and imaging findings. Surgical resection remains the cornerstone of treatment, with complete resection offering better outcomes. Prognosis is poor, particularly with larger tumors, partial resection, and high-grade lesions. Adjuvant therapy's efficacy is uncertain.

**Conclusion:** LMS of the renal vein is a rare entity with challenging diagnosis and management. Radical surgical resection remains the mainstay, but prognosis is guarded, especially in high-risk cases. Further research is needed to optimize treatment strategies for this rare malignancy.

## 1. Introduction

Angiogenic leiomyosarcoma (LMS) is a soft tissue sarcoma, which occurs in the inferior vena cava (IVC) in more than 50% of the cases. LMSs of the renal vein are very rare tumors of the retroperitoneum with only approximately 30 cases reported in the literature [1]. LMSs have a slow growing rhythm and present with nonspecific symptoms which makes their preoperative diagnosis very difficult [2]. We present a case of a primary LMS of the left renal vein.

## 2. Case Report

**Figure 1:**
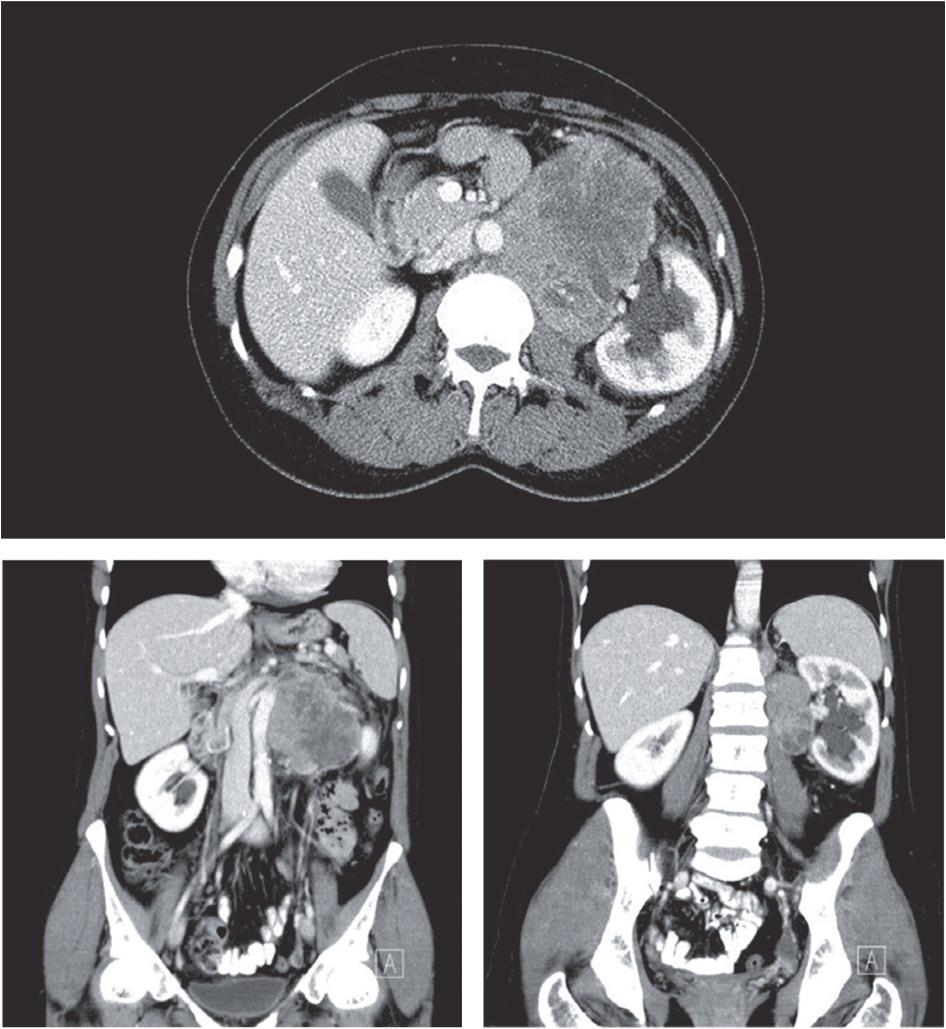
Enhanced computed tomography showing retroperitoneal tumor, interposed between the aorta and the left kidney and axial and coronal plane.

**Figure 2:**
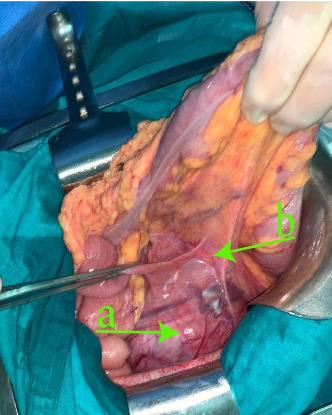
(a) View of the mass inside the abdominal cavity. (b) Angle of Treitz.

**Figure 3:**
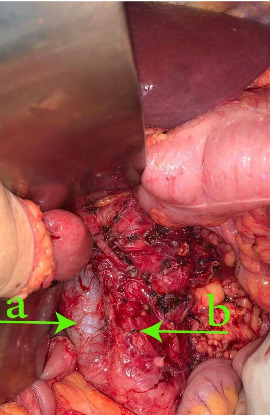
Intraoperative view of the abdomen after extracting the mass. (a) Inferior vena cava. (b) Aorta.

**Figure 4:**
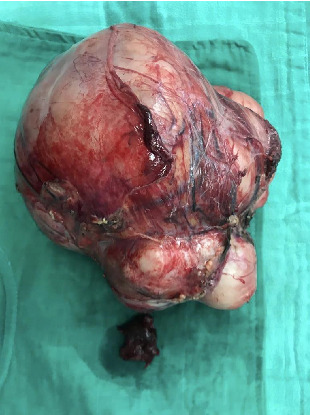
Surgical specimen.

A 73-year-old woman presented with a history of hypertension and a left colon well differentiated adenocarcinoma; she was treated in our department with a laparoscopic left colectomy with colorectal anastomosis in late 2009. In 2022, this patient developed intermittent left flank pain. Clinical examination revealed a voluminous left flank mass. Abdominal ultrasound and CT-scan showed an exophytic and heterogeneously enhancing left latero–aortic mass measuring 140 mm × 100 mm, this mass was located between the aorta and the left kidney (Figure 1). The pathology study on the biopsy of the mass revealed that the morphological aspect with the immunohistochemistry test were compatible with a smooth muscle tumor, but was not decisive on the malignancy. Exploratory laparotomy evidenced a multinodular, solid, and para-aortic 14 cm tumor surrounding the vessels of the left renal hilum (Figures 2 and 3). An en bloc tumor resection was performed with a necessary removal of the left kidney who was not salvageable due to extensive hilum invasion (Figure 4).

The patient's postoperative follow up was unremarkable and she was discharged 5 days after surgery.

**Figure 5:**
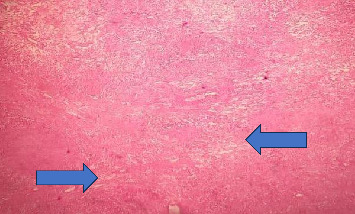
Microscopic view showing a tumor composed of fascicles of spindle-shaped cells (arrows).

**Figure 6:**
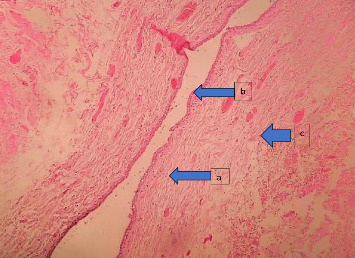
Microscopic view showing a proliferation of spindle cells (a), exhibiting moderate eosinophilic cytoplasm and elongated nuclei with blunt ends (b), displaying variability in nuclear morphology (c) in certain regions.

Pathological examination of the specimen reported a 17 cm tumor compressing the renal parenchyma, there was a capsule separating them which exclude any renal origin. Microscopically, they tend to display bundles of spindle-shaped cells, with flat nuclei and fibrillary appearing cytoplasm. Renal vein LMS originating from the retroperitoneum shows nuclear atypia with mitoses (Figures 5 and 6).

Immunohistochemical study stained positive for caldesmon and desmin. They were negative for cytokeratin AE1/AE3, epithelial membrane antigen (EMA), CD34, melan A, PS100, AML, RO, PR, CD117, Dog, and CDK4. The pathological diagnosis of LMS of the renal vein, grade II of the National Federation of Centers for the Fight Against Cancer (FNCLCC) was, therefore, retained.

Currently our patient is closely followed, 14 months after surgery she is doing well showing no complications and no sign of local or distant recurrence.

## 3. Discussion

LMSs originating from blood vessels are uncommon tumors [3], comprising only 5%–10% of soft tissue sarcomas [4], following liposarcoma and malignant fibrous histiocytoma. These tumors are aggressive and carry a poor prognosis, with vascular structures being an unusual site of origin [5]. Among vascular LMSs, the IVC is the most commonly affected site, accounting for 70% of cases [6]. Primary LMSs of the renal vein are exceptionally rare, with only a handful of cases documented so far [6–8].

These tumors predominantly afflict women and typically manifest in adulthood, with an average age of onset at 57 years. Interestingly, they tend to occur more frequently on the left side [8], which is attributed to its longer length in comparison to the right renal vein [7]. Various theories have been proposed to explain this phenomenon, including hormonal influences such as estrogen stimulation [4], although such theory holds little value in our case since it is a 73 years old menopaused female.

Presentation of renal vein LMSs often occurs at advanced stages, with symptoms developing late in the disease course [9]. In the past, numerous cases were incidentally discovered during autopsies. However, with the advancement of imaging technologies and increased public awareness regarding the importance of early medical consultation, these tumors are now more frequently detected through routine medical examinations [5]. Clinical symptoms vary depending on factors such as tumor location, growth rate, and impact on blood flow. Common symptoms include abdominal pain and weight loss, while hematuria and palpable abdominal masses are less common [3].

Magnetic resonance imaging (MRI) and contrast-enhanced CT scans reveal radiological features that lack specificity, making it challenging to differentially diagnose from other retroperitoneal solid tumors. However, these imaging modalities play a crucial role in surgery planning by delineating the spatial relationship of the mass with adjacent structures [2]. CT scans typically depict a solid and well-defined mass with minimal contrast enhancement around the renal hilum region, while on MRI, a distinct lesion typically appears in the renal hilum, exhibiting an isointense signal relative to the kidney on T1-weighted images, and a slight increase in signal intensity on T2-weighted images [10]. In our case, given its huge size, the tumor extended beyond the region of the left renal hilum and compressed a big portion of the renal parenchyma, which made the diagnosis more challenging.

Approximately half of the cases present a metastatic disease or are unresectable due to local invasion, it can also spread using the hematogenic pathway. Metastasis can affect the liver (25%), lungs (63%), bones (19%), and less frequently, lymph nodes. The definitive diagnosis is made by a pathology study, LMSs contain predominantly monomorphic cells. Microscopically, they present with typical fascicles of smooth muscle cells [11]. They stain positive for caldesmon and desmin in the immunohistochemistry test [12].

The gold standard treatment consists of en bloc surgical resection of the tumor, including nephrectomy. Studies performed at Memorial Sloan Kettering, New York, showed that the major prognostic factor is total surgical resection. When it is complete, 5 years survival free of disease is approximately 60%, vs. just 30%–35% when it is partial [2]. In the international register of Mingoli et al. [13], radical tumor resection was associated with better 5-year survival rates (49.4%). Hines et al. [14] reported that 68% of patients presenting with healthy resection margins survived at 5 years. They determined that surgical resection of the tumor with negative margins has been shown as the most important survival factor for those without widespread metastasis [14].

Little is known about prognostic factors because of lack of large systematic case series, accumulated evidence based on review of previously published reports indicate an overall poor prognosis. The factors that will determine the risk of local recurrence and metastasis of vascular LMSs are the location, mitotic rate, and the size of the tumor (>3 cm) [15]. Another recent study of prognostic factors shows that in univariate analysis, factors predictive of overall survival are surgical margins, while factors predictive of local recurrence free survival are vascular luminal extension and grade. No factors predictive of distant metastasis free survival were identified [16]. Once total removal is performed, the major prognostic factor becomes histological grade, with 5 years free of disease survival of 90%–95% for low grade tumors, and of 30%–35% for high grade tumors [2].

It has been reported that chemotherapy and radiotherapy were ineffective, adjuvant therapy is generally used to high grade tumors, with partial resection [17]. Hines et al. [14] suggests that radiation therapy may prolong survival after surgical resection. Adjuvant radiation therapy and/or chemotherapy may have some benefits in the treatment of LMS of the IVC, but the rareness of LMSs of the renal vein makes proving the efficiency difficult [14].

## 4. Conclusion

LMS of vascular origin is a rare and aggressive pathology. Primary localization at the renal vein is rarely seen. The diagnosis is difficult and can only be made by pathologic features. The gold-standard treatment of LMSs of the renal vein is radical nephrectomy with an en bloc resection of the tumor. Tumors greater than 3 cm, partial resection, unhealthy margins, and high histological grade harbor unfavorable prognosis, increasing the percentage of local recurrence and/or distant metastasis. The appropriate treatment is still controversial given the extreme rarity of this neoplasm.

## Data Availability

The data that support the findings of this study are available from the corresponding author upon reasonable request.
